# Supplementary material for: Evaluating generative artificial intelligence’s limitations in health policy identification and interpretation
Source: PLoS One. 2024 Dec 12;19(12):e0312078. doi: 10.1371/journal.pone.0312078 (PMC11637257; doi:10.1371/journal.pone.0312078)
Supplement: S1 File — (DOCX) [file pone.0312078.s001.docx]

**AMP EID Data Collection and Inclusion Protocol**

*The methodology must address the process of developing subtopics, identifying relevant policies, the criteria used for inclusion of policies, and the mechanism of verification used during the project. Standards for the development of the methodology can be found below.*

*It is imperative that the methodology be updated as you conduct your research, as to accurately reflect the processes followed by the research team and to standardize data collection across the research project. Any significant changes to the methodology during the course of data collection must be documented and approved by the PI before being applied to the project. At the conclusion of data collection for a topic, verify that your methodology has been updated and share the finalized version with the project lead for review.*

1. Methods for the Development of Subtopics

*This section describes the methods and sources used to develop the data taxonomy of your research project.*

1. Literature and Landscape Review
   1. As noted in the SOP, a literature and landscape review must be conducted to inform the development of subtopics. Within the methodology, include a brief description of the role of this process in defining areas of study.
   2. All available resources/databases identified through the landscape review and used to inform the project should be noted here.
2. Consultation with Subject Matter Expert(s) (SME)
   1. In the case that interviews or consultations with SMEs were conducted to verify the relevance of selected subtopics, notation of these meetings should be included.
3. Additional Resources Used in the Development of Subtopics
   1. All other resources consulted or otherwise used to shape subtopics should be noted in this section.  Where relevant, links or pdfs should be saved in the project folder.

1. Methods for the Identification of Relevant Policies and Verification Mechanisms

*This section describes the methods for surfacing relevant policies, the process for their capture, and the mechanism developed to verify that all potentially relevant policies from a country had been reviewed. The Proof of Concept exercise will also be described in this section.*

1. Sources/Existing Repository Use
   - 1. The use of existing policy repositories for the collection of relevant documents is acceptable, and at times, inevitable for this project.  This is a critical place to start. However, it is paramount that researchers utilize available repositories solely as a starting point, mindful to uphold principles of academic integrity and add value with their work. As such, any policy surfaced through the use of an existing repository should be acknowledged and verified by rigorously searching for an original copy of the document from the authoring country’s domain. Refer to “Data Entry in Airtable”, sections 1.b.5 and 1.b.6 for more information on entering these documents into Airtable. Within the methodology, databases or policy repositories utilized for surfacing policies from multiple countries must be noted in this section. Likewise, general methods for finding the policy on a country’s top-level domain should also be included here. Questions that arise on the appropriate use of existing repositories should be brought to the attention of the PI.
2. Methods for Searching for Digitized Policies
   - 1. A **standardized** method of searching for relevant policies must be developed and documented before the research project begins. As appropriate, a list of 3 query terms should be developed for each subtopic and used to search for policies for each country. Before conducting the proof of concept study, a preliminary list of 5 query terms must be developed. At the conclusion of the proof of concept exercise, the finalized series of the 3 most effective search queries should be included in your methods section and should again be reviewed for accuracy at the conclusion of data collection. This series of search terms will act as a preliminary verification method, as failing to surface appropriate policies after conducting a standardized method of searching will result in the determination that the country does not have an applicable policy available for inclusion in the project.
3. Proof of Concept [10 Country Sample Study]
   - 1. The Proof of Concept exercise will facilitate an assessment of the feasibility and appropriateness of the proposed subtopics and methods for policy identification.
     2. Researchers should begin by selecting an array of 10 countries representative of a variety of WHO regions, income levels and political situations. Questions about appropriate countries for this exercise should be directed to the Project Manager or PI.
     3. The researcher should then begin data collection for the 10 test countries, documenting which of the search terms surface the most applicable policies, and noting patterns that arise within the policies identified for each subtopic.
     4. After completing data collection for the proof of concept exercise, the researcher should review the search queries and determine which proved to be the most appropriate. The researcher should also use this sample of collected policies to determine inclusion criteria.
4. Methodology for Policy Review and Documentation of Inclusion Criteria
   1. Policy Review Methods
      1. To a reasonable extent, the text of the policy should be reviewed to determine its applicability to the subtopic and assess if it meets the inclusion criteria.
      2. For policies written in a language that is not spoken/read by the researcher, the use of Google Translate or a similar language translation service is appropriate. Moreover, reasonable consultation of contacts with expertise in foreign languages is appropriate when translating software is not sufficient for the policy analysis.  Please contact the Project Manager if you need assistance. Note the way(s) in which policies were translated for review.
   2. Development of Inclusion Criteria
      1. Policies included in the Analysis and Mapping of Policies for Emerging Infectious Disease database are required to be legally binding. Strategies, plans and other documents outlining future actions of the national government or suggesting criteria for the development of legally-binding policies should be excluded from this research effort. A full list of policy categorizations can be found in the glossary. Note the criteria for including various document types in your methods.
      2. For each subtopic, the researcher must develop specific criteria for the inclusion of policies. For subtopics with more than two statuses, methods should include details on the qualifications for inclusion within each status. While these criteria should be generalizable across countries, they should also be replicable, allowing another researcher to distinguish between the inclusion criteria for each status. Within the methods, specific criteria for inclusion in each status should be documented under each subtopic.
      3. Before conducting the Proof of Concept exercise, inclusion criteria should be loosely defined. After the proof of concept and consultation with PI, inclusion criteria should be solidified and universally applied to the research project.
